# Editorial: Affective shared perception

**DOI:** 10.3389/fnint.2022.1024267

**Published:** 2022-09-09

**Authors:** Alessandra Sciutti, Pablo Barros, Ginevra Castellano, Yukie Nagai

**Affiliations:** ^1^Cognitive Architecture for Collaborative Technologies (CONTACT) Unit, Italian Institute of Technology, Genoa, Italy; ^2^Uppsala Social Robotics Lab, Department of Information Technology, Uppsala University, Uppsala, Sweden; ^3^International Research Center for Neurointelligence, The University of Tokyo, Tokyo, Japan

**Keywords:** affective computing, perception, shared perception, developmental learning, machine learning, human-robot interaction

In everyday life, we assume that we perceive accurately the reality surrounding us, similarly to what a camera installed on a robot would capture. This assumption is, however, far from correct: our perception is an inferential process, where our brain integrates the inputs coming from the senses with prior knowledge (Jazayeri and Shadlen, 2010) and “colors” it as a function of our internal state (Dominguez Borras and Vuilleumier, 2013). As a result, two individuals observing the same physical event might perceive two different realities. The importance of context and past experiences has particular implications for affective understanding. Humans indeed adapt affective perception as a function of the current stimulation, the context, the history of the interaction, and the partner's status (Lee et al., 2012; Krautheim et al., 2019). This adaptation influences their behavior, modifying the social and affective perception of both partners and the evolution of the interaction.

The asymmetry in perception is even more striking in human-robot interaction, as machines still miss this inferential and affective modulation mechanism (Figure 1).

**Figure 1:**
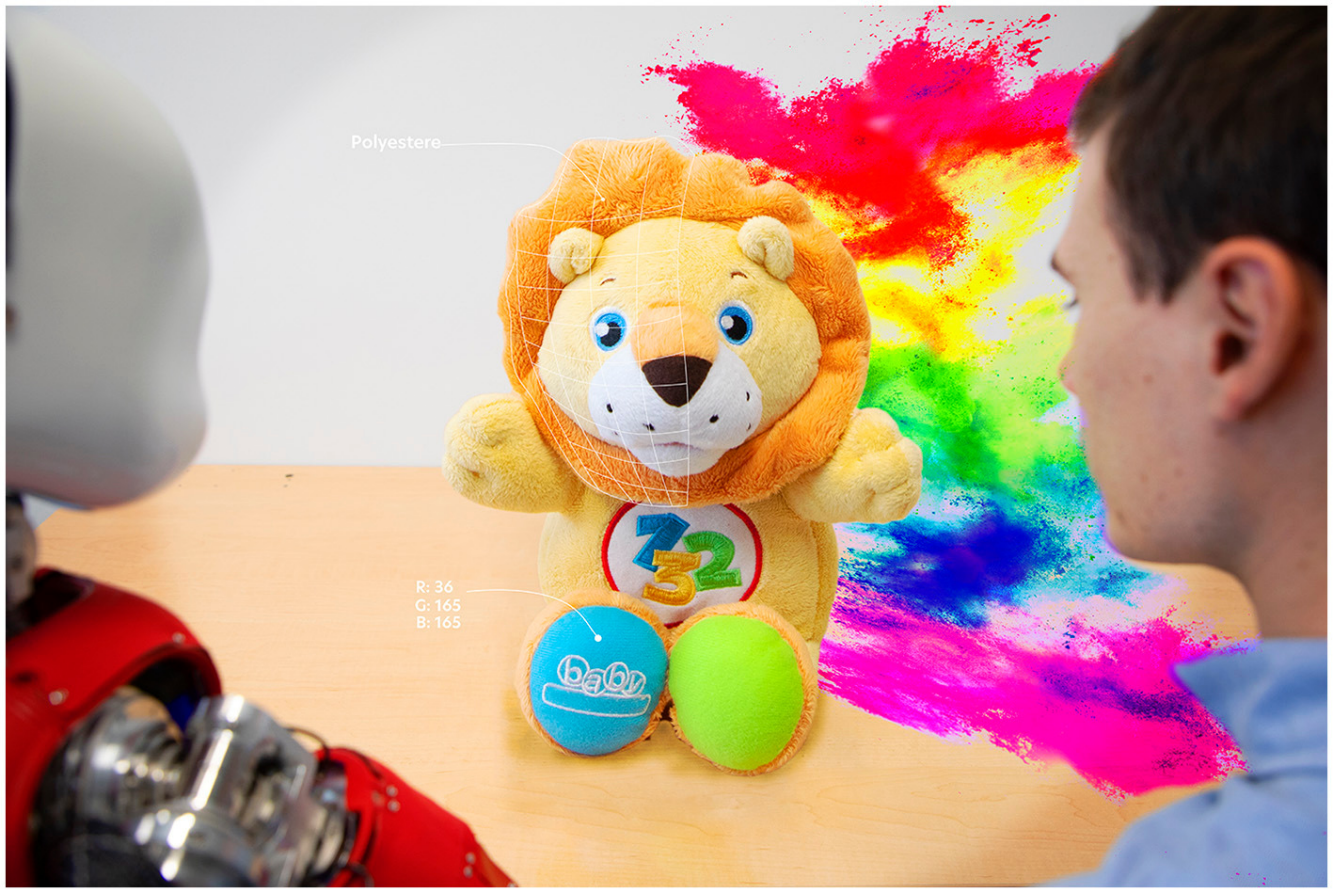
Each individual's perception results from an inferential process, where prior experience and affective states shape how we perceive others and our environment. Establishing a shared perception with another agent, be it a human or a robot, requires consideration of how the other is processing the information from the environment. Image credits: Laura Taverna, Italian Institute of Technology.

However, in human-human interaction, we often observe mutual understanding, suggesting that it is possible to establish shared perception. Recent evidence shows that the brain tends to reduce the reliance on prior experience when someone is involved in an interaction in favor of a more veridical perception of the physical event (Mazzola et al., 2022; Tsfasman et al., 2022). Extending our comprehension of the processes by which our brain enables sharing perception with another agent is a significant challenge in building computational models promoting effective human-machine understanding.

Most current research on modeling affective behavior does not consider such dynamic shared perception. Several contributions focus on pre-trained learning models (Li and Deng, 2020), which are purely data-driven, or on reproducing existing human behavior into computational models (Revina and Emmanuel, 2021). Such methods allow for easily reproducible solutions but also often limit the generalizability of the results to specific and relatively simple situations. Understanding shared perception as part of affective processing would advance the field toward a real-world affective computing system.

This Research Topic collects for the first time contributions addressing the issue of affective shared perception from a multi-disciplinary perspective.

Manzi et al. (*Can you activate me? From robots to human brain*) discuss under which conditions the perception of robots as social partners leverage the same social brain areas as human perception. Starting from existing literature, they identify the agents' anthropomorphism and their actions (type and kinematics) as crucial factors and suggest that prior experience with the actual limits of current robotics platforms might inhibit the automatic social activation.

On a related topic, Perugia et al. (*Does the goal matter? Emotion recognition tasks can change the social value of facial mimicry toward artificial agents*) investigate how the embodiment and human-likeness of an artificial agent can affect people's mimicry of its facial expressions. Spontaneous facial mimicry appeared when participants were less sure of the recognized emotions. Moreover, participants mimicked the least the agent that they perceived as the most anthropomorphic and likable, suggesting that these factors do not necessarily favor mimicry.

These two works show that human internal models can automatically be “reused” to sustain human-agent interaction. Also, artificial agents should be endowed with the skill to adapt and understand humans.

In this framework, Hughson et al. (*Investigating the role of culture on negative emotion expressions in the wild*) characterize a limitation of current approaches for emotion recognition, which fail to account for the role of culture in the expression of negative emotions. The authors focused on contempt, anger, and disgust expressed by persons of North American, Persian, and Filipino cultures. Their analysis identified the specific social signals differing across those cultures and highlighted how automatic classification methods performance significantly decreased when comparing the classification of North American expressions to those by Filipino and Persian.

Hemeren et al. (*Kinematic-based classification of social gestures and grasping by humans and machine learning techniques*) propose a direct comparison between human and machine learning classification. The authors compare grasping and social gesture recognition performed by humans and four machine learning techniques. The gestures were rated according to the extent they were perceived to be grasping and to be social. Humans rated differently according to the task, whereas the machine learning techniques provided a similar classification of the actions according to grasping kinematics and social quality. Overall, the results showed that human perception relies on kinematic information for perceiving the social aspects and intentions from different actions.

Beyond analyzing human and machine perception differences, it is essential to build models that consider these asymmetries.

Horii and Nagai (*Active inference through energy minimization in multimodal affective human-robot interaction*) focused on resource limitation, a common issue in robotics: a robot cannot always deal with all the available modalities simultaneously. In understanding the partner's emotional state, for instance, a robot should select the most informative modalities among those available to estimate the target states. To this aim, they propose an active perception method based on energy minimization. They demonstrate that the proposed approach improves accuracy using limited information in affective human-robot interaction.

Matarese et al. (*Perception is only real when shared: A mathematical model for collaborative shared perception in human-robot interaction*) considered instead an interaction in which the robot and the human have different access to information about the task at hand. They proposed a model to overcome the asymmetries in perception and validated it in a collaborative Lego game. They demonstrated that a robot giving suggestions by considering the partners' point of view and using its inference about their common ground to select the most informative hint led to better joint performances. However, this behavior did not significantly improve participants' subjective evaluation of the robot.

A few authors explored how to model the past and current experience in the robot and use that as a context, modulating perception and action selection.

Khan and Cañamero (*The long-term efficacy of “social buffering” in artificial social agents: Contextual affective perception matters*) simulated a small society of artificial agents whose goal was to “survive” by maintaining the stability of their internal environment through physical and social behaviors. They modeled a few hormonal mechanisms, serving as “physiological biomarkers” and encoding dynamically historical and current environmental information. They found that hormonally-mediated, social-based stress-regulating effects provided significant advantages to the short-term wellbeing of agents with affective, social support. A fascinating insight from this work is that hormonal mechanisms can act as functional affective states, which synthesize multiple sources of information into a small subset of embodied signals that can play an adaptive and predictive role in embodied, socially-adaptive agent models.

Churamani et al. (*Affect-driven learning of robot behavior for collaborative human-robot interactions*) propose a novel framework for affect-driven behavior generation in social robots. The partner's behavior influences the robot's evolving affective representation. The latter is also shaped by the robot's affective and behavioral disposition, which influences the learning process of responding to the partner's actions. Hence, the robot shares a common view of the interaction with the partner but reacts originally without mimicking their behavior. In an experimental validation, consisting of a set of Ultimatum Games with the robot NICO, the authors demonstrate that the robot's intrinsic mood, modulated by its affective dispositions and history with the partner, governs its behavior. Different “moods” lead to different robot perceptions by the human participants.

These researches provide a solid basis for advancing our understanding of affective shared perception. Still, future cross-disciplinary efforts are needed to bridge more deeply the comprehension of human social cognition and the development of cognitive robotics.

## Author contributions

All authors contributed equally to the preparation of the editorial.

## Funding

This work has been supported by a Starting Grant from the European Research Council (ERC) under the European Union's Horizon 2020 research and innovation programme. G.A. No. 804388, wHiSPER.

## Conflict of interest

The authors declare that the research was conducted in the absence of any commercial or financial relationships that could be construed as a potential conflict of interest.

## Publisher's note

All claims expressed in this article are solely those of the authors and do not necessarily represent those of their affiliated organizations, or those of the publisher, the editors and the reviewers. Any product that may be evaluated in this article, or claim that may be made by its manufacturer, is not guaranteed or endorsed by the publisher.
